# Melatonin Treatment Affects Wax Composition and Maintains Storage Quality in ‘Kongxin’ Plum (*Prunus salicina* L. cv) during Postharvest

**DOI:** 10.3390/foods11243972

**Published:** 2022-12-08

**Authors:** Xin Lin, Shian Huang, Donald J. Huber, Qin Zhang, Xuan Wan, Junsen Peng, Dengcan Luo, Xiaoqing Dong, Shouliang Zhu

**Affiliations:** 1 Fruit Crops Center of Guizhou Engineering Research, College of Agricultural, Guizhou University, Guizhou 550025, China; 2 Guiyang Agricultural Reclamation Investment Development Group Co., Ltd., Guizhou 550001, China; 3 Horticultural Sciences Department, Institute of Food and Agricultural Sciences, University of Florida, Gainesville, FL 32611-0690, USA; 4 Guizhou Workstation for Fruit and Vegetables, Guizhou 550025, China

**Keywords:** melatonin, ‘Kongxin’ plum, cuticular wax, morphology, composition, storage quality

## Abstract

Cuticular wax is an essential barrier against biological and abiotic stress and is also an important factor affecting fruit storage quality. This paper investigated the effect of melatonin treatment on cuticular wax and the storage quality of plum fruit at low temperature storage of 4 ± 1 °C. ‘Kongxin’ plum was treated with 150 μmol·L^−1^ melatonin, dried overnight at room temperature 25 ± 1 °C, and then stored at 4 ± 1 °C for 40 d. The microstructure of the fruit epidermis was examined after 0, 20, and 40 d of storage, and the wax composition and fruit storage quality were measured at 10 d intervals. The results demonstrated that melatonin promoted the disintegration and thickening of rod-shaped waxy crystals of ‘Kongxin’ plum fruit and inhibited the combination of disintegrated wax and inner wax. Melatonin maintained fruit firmness and decreased the correlation between fruit firmness and other storage quality parameters. The correlation between firmness and wax composition was enhanced. Melatonin promoted long-chain alkanes that were positively correlated with firmness and water retention and strengthened the correlation between the length of the alkane chain and storage quality parameters but reduced the difference between alkane isomers and storage quality parameters.

## 1. Introduction

The surface of ‘Kongxin’ plum (*Prunus salicina* Lindl cv. ‘Kongxin’) is yellowish-green and has a layer of silvery-white powder. The flesh and stone are naturally separated at maturity. Its flesh is crisp and tender, fragrant and sweet, and juicy, with good taste and bright color. It is also rich in proteins, vitamins, and micronutrients, and is popular with consumers. However, plum fruit quickly loses its commercial value because of its highly perishable nature after harvest, so commercial treatment is necessary to prolong storage [1]. Low-temperature storage is the most common and valuable treatment [2]. In addition, controlled-atmosphere storage, environment-friendly chemical reagents (e.g., 1-methylcyclopropene and ClO_2_), and polyvinyl-chloride (PVC) packaging are used for plum-fruit storage to maintain the usual color and flavor of the fruit flesh [3,4,5,6,7]. Some studies have reported that exogenous hormones can improve the tolerance of plum fruit under low-temperature storage and maintain fruit quality while reducing chilling-injury symptoms [8,9,10].

Melatonin (*N*-acetyl-5-methoxytryptamine, MT), one of the most widely studied exogenous hormones in recent years, is a critical tryptophan indole that is widely distributed in organisms. It plays an excellent role in postharvest storage and preservation [11,12,13]. The research on melatonin in fruit storage has mainly focused on fruit quality [14,15]. Exogenous melatonin can promote the synthesis of endogenous melatonin in tomato fruit [16]. Moreover, some studies have reported that melatonin can delay fruit browning and senescence by affecting enzyme activity, regulating membrane lipid and energy metabolism, and triggering a series of changes in metabolism and gene expression to improve fruit quality further [13,17,18,19]. MT may enhance the sustainability of postharvest fruit production systems by facilitating the accumulation of blueberry cuticular wax [20]. However, few studies have addressed the influence of melatonin treatment on the cuticular wax of plum fruit.

Fruit cuticular waxes are waxy crystals with particular structures and amorphous intracuticular wax. The wax components include aliphatic compounds, cyclic compounds, and sterols [21]. Waxes are conducive to protecting fruit from biotic and abiotic stresses [22,23]. Changes in the wax structure and composition of the fruit influence the ability of the fruit to resist the external environment and reduce the storage quality of the fruit [24]. The apple epidermis mainly consists of waxy crystals with lamellar structure and the distribution of reticular or strip microcracks [25]. Apple waxes consist primarily of odd carbon alkanes, and the content of nonacosane is dominant in aliphatic compounds. The alkane content is positively correlated with weight-loss rate and fruit firmness; preharvest application of sprayable 1-MCP can regulate pre- and postharvest apple fruit quality [26,27] and the degradation of nonacosane-protected fruit quality. Nonacosane and octadecanaldehyde positively affected the quality of pear fruit, and olefin and fatty acids had a significant negative correlation with hardness and a significant positive correlation with decay rate [28,29]. The waxy crystals of most citrus fruit are presented as platelet structures. Alkanes and aldehydes are essential substances for forming platelet-shaped wax crystals in the citrus epidermis, and alkanes and triterpenes strongly influence water ingress through the fruit cuticle [30,31]. Dense tubular waxy crystals are distributed to the outer epidermis of blueberry fruit. Triterpenoids and β-diketones are the most abundant components in blueberry waxes, and weight loss of blueberry fruit is determined by the contents of alkane, primary alcohols, and fatty acids [32,33]. However, little is known about the structure and components of plum-peel wax and the effect of wax components on fruit storage quality during postharvest storage.

This study aimed to investigate the effects of melatonin treatment on the epidermal microstructure, composition, and storage quality of ‘Kongxin’ plum fruit, and to analyze the correlation between wax composition and the storage quality of fruit. The results will improve our understanding of wax changes and the storage of plum fruit after harvest and provide reliable information for further research and development of ‘Kongxin’ plum edible wax coatings.

## 2. Materials and Methods

### 2.1. Plant Materials

The ‘Kongxin’ plums (*Prunus salicina* Lindl cv. ‘Kongxin’) with a firmness of approximately 5.70 newtons (5.70 N) and total soluble solids (TSS) of approximately 9.6% were collected from a farmer’s cooperative orchard in Yanhe County, Guizhou Province (108.52° E, 25.37° N), China, on 12 July 2020. The fruit tree was planted in the fall and winter of 2003. The space was 4 m × 5 m for grafting rootstock to wild peach, and all fruit were collected from the canopy outside the sun. Within four hours of harvest, the fruits were transported to the laboratory at the Department of Horticulture, College of Agronomy, Guizhou University. Plum fruit with uniform size and color and without any evident damage were selected for treatment.

The fruit were randomly divided into two groups. Each group contained three replicates, with approximately 200 plums per replicate. One group was immersed in a solution of 150 μmol·L^−1^ melatonin (MT) for 20 min at ambient temperature, and the control samples were treated with distilled water for 20 min at ambient temperature. The fruit was dried overnight at room temperature of 25 ± 1 °C and then placed into cartons and placed in cold storage at 4 ± 1 °C under 90% relative humidity for 40 days (40 d). Sampling and analyses took place on days 0, 10, 20, 30, and 40 of storage. The fruits stored for 0, 20, and 40 d were selected for the appearance and microstructure-of-plum-peel analysis. All samples were promptly frozen in liquid nitrogen and stored in a −80 °C ultra-low temperature refrigerator (Thermo Fisher Scientific, Suzhou, China).

### 2.2. Observation of Peel Appearance and Microstructure

Each treatment (MT or control) was analyzed with ten fruits for replication (containing three replicates). Peel sections (3 mm × 3 mm × 2 mm) were taken from three points of each fruit at the equator (opposite the navel, on both sides). The wax structure of the peel was observed with a microscope. Peel sections were fixed in 500 μL 2.5% glutaraldehyde overnight. Tissue was transferred to 1 mL of 2.0% phosphate buffer (PBS, pH 7.0) and soaked for 3 min. Gradient tert-butanol (50%, 70%, 90%) was used for dehydration for 10 min successively, and finally 100% tert-butanol was used for dehydration twice for 10 min each. The sample after dehydration was vacuum-dried by freeze drier (LGJ-10D, Beijing China). After the ice crystal volatilized, the sample was placed on the bonding table and sprayed with gold by an ion-sputtering coater (E-1010, KYKY, Beijing, China), and the microstructure was observed on a scanning electron microscope (S-3400N, Hitachi, Japan).

### 2.3. Extraction of Cuticular Wax

Cuticular wax was extracted using the method of Dong et al. [26], with modifications. Specifically, each group randomly selected ten plums (containing three replicates) and entirely immersed them in 500 mL beakers containing 300 mL chloroform/methanol (4:1, *v*/*v*). The beakers were placed in a water bath at 40 °C, and the fruit was stirred for 30 s. Each fruit was extracted three times. The extracting solution was transferred to a round-bottom flask, concentrated at 40 °C using a rotary evaporator, and transferred to a brown bottle (40 mL). The wax extract was then dried with a stream of nitrogen gas and stored at −80 °C for subsequent gas chromatography-mass spectrometry (GC-MS) analysis.

### 2.4. Chemical Analysis of Cuticular Wax

The samples stored at −80 °C were redissolved in 10 mL chloroform, followed by 1 min of vortex and 5 min of ultrasound. An aliquot (2.5 mL) of the redissolved solution was transferred to a 4 mL glass bottle, and the wax extract was blown dry with liquid nitrogen. Chloroform (500 μL) and 80 μL N,O-bis (trimethylsilyl) trifluoroacetamide (BSTFA) reagent (containing 1% Chlorotrime-thylsilane (TMCS)) were added into a 4 mL glass bottle filled with the nitrogen-dried sample. The samples were then vortexed for 1 min and subjected to ultrasound for 5 min, then reacted at 70 °C for 1 h. The samples were placed at room temperature for 30 min followed by centrifugation at 12,000× *g* for 10 min. Two hundred mL of supernatant was transferred to a lined glass bottle for GC-MS metabolomics analysis. A mixed standard of n-alkanes (C7-C40, 20 mg·L^−1^) was transferred to a glass bottle with a lined tube for GC-MS metabolomic analysis.

GC-MS analysis was carried out using DB-5MS capillary column (30 m × 0.25 mm × 0.25 μm, Agilent J&W Scientific, Folsom, CA, USA) with ultra-pure helium (purity not less than 99.999%) as carrier gas at a flow rate of 1.0 mL·min^−1^. The inlet temperature was 260 °C. The heating procedure was as follows: the initial temperature was 80 °C, rising to 200 °C at 10 °C·min^−1^ for 2 min, rising to 260 °C at 15 °C·min^−1^, and finally rising to 315 °C at 5 °C·min^−1^ for 10 min.

The mass spectrometry conditions were as follows: an electron-bombardment ion source (EI), ion-source temperature of 230 °C, quadrupole temperature of 150 °C, and electron energy set to 70 eV. The scanning mode was full-scan mode (SCAN) and the quality scan ranged from 50 to 650 m·z^−1^. The wax components were qualitatively analyzed by the NIST database (https://webbook.nist.gov/chemistry/ (accessed on 12 April 2021)) or by comparing the mass spectrum and retention time of the component substances with the retention time for qualitative determination and an internal standard with available content-determined quantification of the wax composition. The components with more than 80 components were selected as the final results [34].

### 2.5. Determination of Fruit Firmness, Weight Loss, TSS, and Titratable Acid (TA) Content

Fruit firmness was measured using a digital readout fruit pressure tester (GY-4, Handpi Industry Co. Ltd., Yueqing, China) with a 3.5 mm diameter cylindrical probe (three replicates of 30 plums). The fruit’s two symmetrical locations at the equator of the fruit were peeled (about 1 mm thick) to measure the fruit firmness, and each location was pressed for 5 mm. The results were expressed in newtons (N).

Weight loss (%) was calculated using the formula (%) = [(M0 − M1)/M0] × 100, where M0 and M1 represent the initial weight of each plum and the measured weight of each plum during storage, respectively.

The TSS and TA were measured using a hand-held digital refractometer (Model PAL-BX/ACID1, Atago Co. Ltd., Tokyo, Japan) and dripping the fruit juice into the sample slot to determine the value of the TSS, followed by diluting the juice with distilled water 50 times to calculate the value of the TA, and the values were expressed as Brix (percent) and percentage, respectively. The values of firmness, TSS, and TA were determined in triplicate [35].

### 2.6. Determination of Soluble Sugar and Soluble Protein Content

The soluble sugar content was determined by anthrone colorimetry [36]. In brief, this method was carried out according to the following steps: place fruit flesh tissue (0.2 g) into a test tube, add 10 mL of distilled water, seal it with plastic sealing film, extract it twice in boiling water for 30 min, filter the extract into a 25 mL volumetric flask, rinse the test tube and residue repeatedly, and fix the volume to the scale. Suck 0.5 mL of sample extract, 1.5 mL of distilled water, 0.5 mL of anthrone ethyl acetate reagent, and 5 mL of concentrated sulfuric acid. Thoroughly shake, immediately put the test tube into a boiling-water bath, keep it warm for 1 min, take it out, and naturally cool it to room temperature. The absorbance of the solution was measured at 630 nm, and the results were expressed as a mass fraction (%).

The content of soluble protein in plum flesh was determined according to the method described by Cao et al., (2007). Flesh tissue (1.0 g) was homogenized on ice and extracted using 5 mL of pre-cooled phosphate buffer (PBS, 50 mmol·L^−1^, pH 7.8). After centrifuging at 12,000× *g* at 4 °C for 20 min, the supernatant was collected as a soluble protein extract and was cryopreserved. Then, we took 1 mL of supernatant, put it into the plug tube, added 5 mL of coomassie bright blue G-250 solution, and thoroughly mixed the solution and placed it for 2 min. The absorbance of the solution was measured at 595 nm. The results were expressed as soluble protein content per gram of fruit (g·kg^−1^).

### 2.7. Determination of Pectin Substance and Cellulose Content

The protopectin (PP) and soluble pectin (SP) contents were determined by the methods of He et al., with some modifications [37]. In brief, 1.0 g sample of tissue was dissolved using 10 mL of 95% ethanol (*v*/*v*), after which the sample was heated in a boiling-water bath for 30 min while being stirred. After cooling, the sample was centrifuged at 10,000× *g* for 10 min, and the supernatant was then discarded. The pellets were washed twice with hot 95% ethanol (*v*/*v*), after which they were resuspended in 25 mL of distilled water; the mixture was then heated at 50 °C for 30 min. Then, the mixture was centrifuged at 10,000× *g*. The supernatant was used to measure soluble pectin (SP), and the pellets were dissolved in 25 mL of 0.5 mol·L^−1^ H_2_SO_4_ and then heated in a boiling-water bath for 1 h. The supernatant was used to measure protopectin (PP). One mL of PP or SP extract was added to 6 mL of H_2_SO_4_ for 1 h in boiling water. After the mixture was cooled, 1 mL of 1.5 g·L^−1^ carbazole reagent was added, and the mixture was then incubated in darkness for 30 min. The sample was measured at 530 nm, and galacturonic acid was used as the standard. The results were expressed by the mass fraction (%) of galacturonic acid generated, respectively.

The ion-bound pectin (ISP) and covalently bound pectin (CSP) were, respectively, extracted from samples using the protocol of the ISP and CSP content kit (Cominbio Biotechnology, Suzhou, China). The absorbance of the sample solution was measured at 530 nm, and the results were expressed as ISP and CSP per gram of fruit (g·kg^−1^).

Cellulose was extracted and measured by the method described by Bu et al. [38], with modifications. Approximately 1.0 g of tissue was ground in a mortar and dissolved with 2.0 mL of acetic/nitric reagent (80% acetic acid: concentrated nitric acid, 10:1 *v*/*v*) and heated in a boiling-water bath at 100 °C for 30 min, centrifuged for 20 min at 4000× *g*. The supernatant was discarded after cooling. The residue was washed twice with distilled water. The washed residue was dissolved in 10 mL of 67% sulfuric acid, mixed well, and diluted to 100 mL with distilled water. One mL of the solution was pipetted into a glass test tube, and then 4 mL of deionized water and 10 mL of cold anthrone reagent (0.2 g anthrone in 100 mL concentrated sulfuric acid) were added and mixed. The tubes were placed in a boiling-water bath for 10 min. After cooling in an ice bath for 5 min, the absorbance of the sample solution was measured at 620 nm and calculated using the purified cellulose standard curve. The results were expressed in mass fraction (g·kg^−1^).

### 2.8. Determination of Polygalacturonase (PG), Cellulase (Cx), and β-Glucosidase (β-GC) Activity Assay

PG and CX were determined using 3,5-dinitro salicylic acid, and β-GC was determined using p-nitrophenyl glucoside hydrolysis. In short, 1.0 g sample of tissue was dissolved using 10 mL of 95% ethanol (*v*/*v*), then placed at a low temperature for 10 min, the sample was centrifuged at 12,000× *g* at 4 °C for 20 min, and the supernatant was then discarded. The precipitate was washed once with 10 mL precool 80% ethanol (*v*/*v*), standing for 10 min. The supernatant was then discarded after centrifugation under the same conditions. The precipitate was added with 5 mL of pre-cooled acetic acid sodium acetate buffer (50 mmol·L^−1^, pH5.5, containing 1.8 mol·L^−1^), and the supernatant was stored at 4 °C for 20 min. After centrifugation, the supernatant was collected as an enzyme extract.

PG, Cx, and β-GC were, respectively, extracted from samples using the PG, Cx, and β-GC activity detection kit (Solarbio, Beijing, China). The activities of PG, Cx, and β-GC were determined according to the manuals in the kit. To determine the activity of PG, 25 μL of enzyme extract, 50 μL of acetic acid-sodium acetate, and 50 mL of 10 g·L^−1^ of polygalacturonic acid solution were added to a 1.5 mL centrifuge tube, respectively, and then mixed and reacted in a 40 °C water bath for 2 h. After being cooled to room temperature, 125 mL of 3,5-dinitrosalicylic acid solution was added to the water bath for 5 min, cooled to room temperature, and then the light absorption value of 540 nm was measured. The activity of PG was represented by unit: one unit (U·g^−1^) of PG activity is defined as 1 μmol galacturonic acid generated per gram of fruit per hour.

To determine the activity of Cx, we added 0.5 mL of enzyme extract into 1.5 mL of 10 g·L^−1^ sodium carboxymethyl cellulose solution, and placed it in a 37 °C water bath for 1 h. After taking it out, we quickly added a 1.5 mL 3,5-dinitrosalicylic acid solution, heated it in boiling water for 5 min, cooled it to room temperature, added distilled water to the constant volume of 25 mL, and measured the absorbance at 540 nm. The determination of enzymatic activity was expressed in units: one unit (U·g^−1^) of Cx activity is defined as 1 μg glucose generated per gram of fruit per minute [39,40].

To determine the activity of β-GC, 0.5 mL p-nitrobenzene-β-glucopyranoside (5 mmol·L^−1^) was added to 0.5 mL enzyme solution and kept at 37 °C for 30 min. Immediately after extraction, 2 mL 1 mol·L^−1^ Na_2_CO_3_ was added to stop the reaction, and the absorbance of the reaction solution at 400 nm was determined. The β-GC activity was quantified by the increase in absorbance at 400 nm, which was caused by the release of p-nitrophenol. The determination of enzymatic activity was expressed in units: one unit (U·g^−1^) of β-GC activity is defined as 1 nmol p-nitrophenol generated per gram of fruit per hour [41].

### 2.9. Statistical Analysis

All experiments were performed with at least 3 replicates. Principal component analysis (PCA) and orthogonal partial least-squares discriminant analysis (OPLS-DA) were performed by SIMCA (V14.1). Statistical data of SPSS19.0 and Duncan’s multi-range test (*p* ≤ 0.05) were used to analyze the storage quality data, and then Origin Pro 2021 was used to draw the line chart. The Correlation Plot of Origin Pro 2021 was used for the correlation heat map.

## 3. Results

### 3.1. Fruit Appearance and Peel Microstructure during Storage

**Figure 1:**
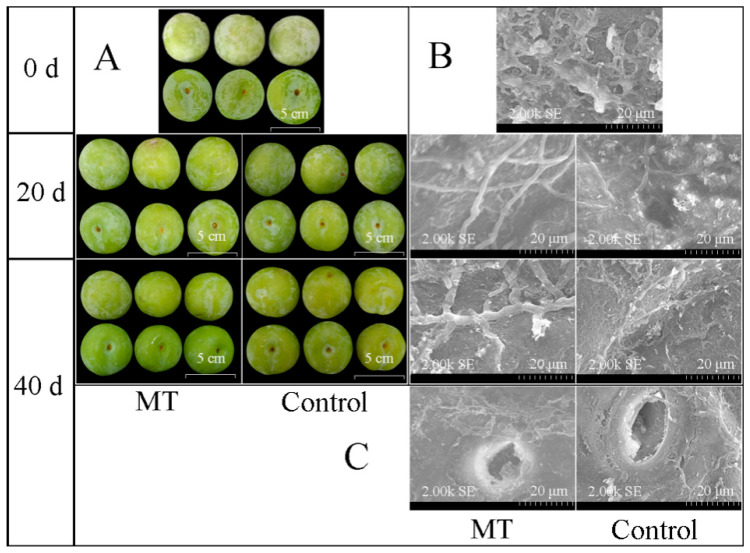
Melatonin treatment affects ‘Kongxin’ plum fruit appearance and peel microstructure during low-temperature storage. (**A**) fruit appearance at 0, 20 and 40 d; (**B**) peel microstructure at 0, 20, and 40 d; (**C**) fruit lenticels at 40 d.

During storage, fruit shrinkage and a gradual reduction of wax bloom were observed. It was observed that the shrinkage degree of MT was lighter than that of the control. The wax frost of the control was more evident than that of MT (Figure 1A). A large number of rod-shaped and flocculent wax structures were aggregated on the peel at 0 d (Figure 1B). In contrast, the characteristic rod-shaped waxy crystals gradually disintegrated and combined with the internal waxes during storage (Figure 1B). Moreover, compared with 20 d rod-shaped wax crystals, 40 d rod-shaped wax crystals appeared thicker in MT due to the disintegration of wax crystals (Figure 1B). Compared with the control fruit lenticels, the fruit lenticels of MT were partially covered by gradually disintegrating waxy crystals at 40 d (Figure 1C).

### 3.2. Cuticular Wax

#### 3.2.1. Multivariate Analysis of Cuticular Wax during Storage

**Figure 2:**
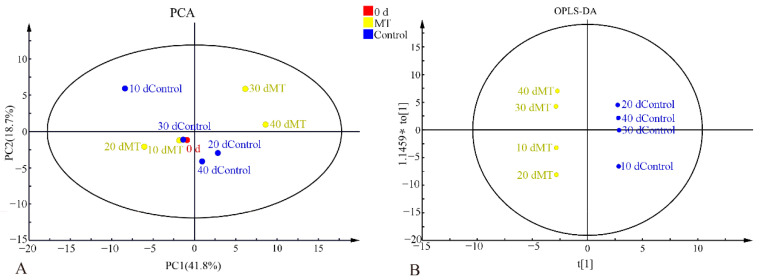
PCA analysis (**A**) and OPLS-DA analysis (**B**) of wax components.

Data-independent MS/MS deconvolution for comprehensive metabolome analysis (MS-DIAL) processed the imported data, and the data with a similar value greater than 80 from 652 metabolites were selected for analysis. Then, 70 wax components were screened to analyze the relative content of the wax components. The relative contents of 70 waxy components were analyzed by PCA (Figure 2A). The first principal component score was 41.8% and the second principal component score was 21.8%. Among the results of the PCA analysis, the MT for 10 d and the control for 30 d were similar to 0 d. Compared with the components for 20 d, the MT components for 30 d changed, and the results of the PCA for 30 and 40 d were in the first quadrant. The components of the control were changed during storage. The wax components of MT and the control stored for 10–40 d were separated by OPLS-DA (Figure 2B). The wax components of MT and the control were compared every 10 d, and the wax of the two groups was separated at 20 and 30 d.

**Figure 3:**
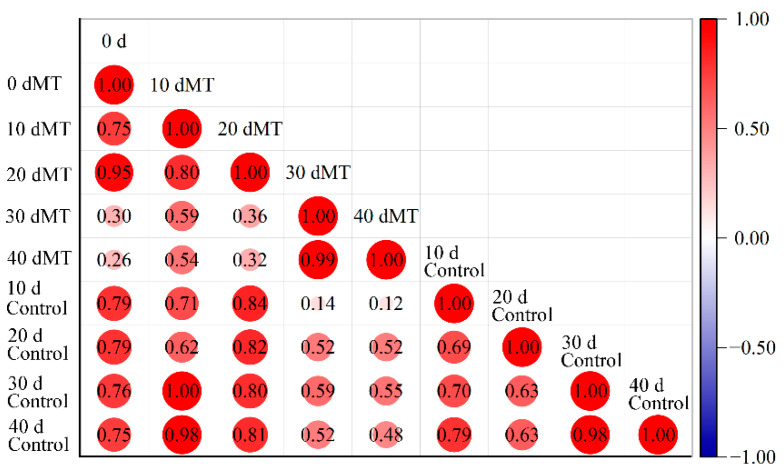
Melatonin affects the correlation of cuticular wax.

Correlation analysis was carried out on the wax components of ‘Kongxin’ pl¾um at each stage to analyze the relationship and influence of melatonin on the changes of wax components during storage (Figure 3). Based on the correlation coefficient, the correlation degree was divided into the following four cases: |r| ≥ 0.8, high correlation; 0.5 ≤ |r| < 0.8, moderate correlation; 0.3 ≤ |r| < 0.5, weak correlation; |r| < 0.3, extremely weak correlation. The wax composition at 0 d was moderately correlated with MT and the control at 10 d. The wax components of MT at 10 and 20 d were highly correlated, and that of the control was moderately correlated. The wax components of MT at 20 and 30 d were weakly correlated, and that of the control was moderately correlated. The wax components of MT at 30 and 40 d were highly correlated, which was the same as that of the control. The correlation coefficients of the wax components during storage of MT and the control were greater than 0, and the wax components were positively correlated with each other. The correlation coefficients between 0 d and the control were 0.5 ≤ |r| < 0.8, with moderate correlation. The correlation coefficient between 0 d and MT changed from moderate correlation and high correlation to weak correlation. In summary, the wax of ‘Kongxin’ plum cuticular wax was altered by melatonin during storage, the wax composition remained stable after the transition period of 20–30 d, and the results of PCA and OPLS-DA were also verified.

#### 3.2.2. Cuticular Wax Composition Content

**Figure 4:**
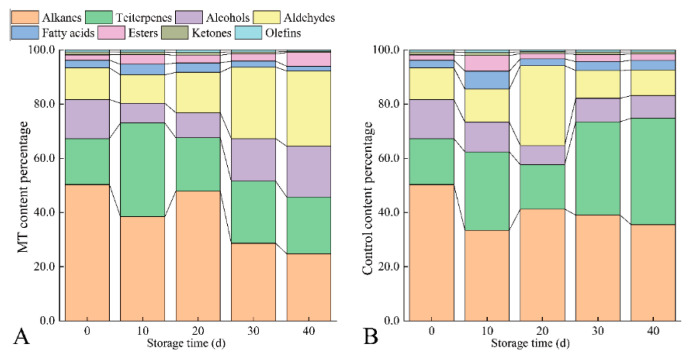
Melatonin treatment affects the proportion of cuticular wax in ‘Kongxin’ plum during storage. The proportion of melatonin group (**A**) and control group (**B**) cuticular wax changed during storage.

The wax composition contents remained unchanged during storage and included 17 alkanes (C12-C36), three triterpenes, 12 alcohols (C12-C28), six aldehydes (C9-C30), 16 fatty acids (C7-C24), 11 esters (C11-C36), 3 ketones (C10-C29), and two olefins (Tables S1 and S2). The content of alkanes in wax accounted for 50.3% at 0 d, which was the most abundant wax component. The relative content of alkanes increased slightly at 20 d and then gradually decreased, and the decline was more significant in MT than in the control (Figure 4A,B). Triterpenes were the second-most-abundant component, accounting for 17.0% at 0 d. The relative content of triterpenes in MT was highest at 10 d and remained stable after 20 d. The content of triterpenes in the control was similar to that in MT at 0–20 d, but the relative content gradually increased after that. Triterpenes became the most abundant component in the control wax at 40 d. Before GC-MS detection, alcohols were converted into corresponding TMS components by the silylation reagent BSTFA, and TMCS components were converted into corresponding alcohol mass during data analysis. The content of alcohols in 0 d wax was lower than that of alkanes and triterpenes, accounting for 14.4%. The relative content of alcohols in MT decreased to the lowest value at 10 d, followed by an increase with further storage. The relative content of alcohols in the control decreased continuously during storage. Because only the data with a similar value more excellent than 80 were selected for analysis, some primary alcohols with high content were not within the analysis range. The relative content of aldehydes in wax remained stable after 30 d. The ester content of MT increased from 2.0% at 0 d to 5.1% at 40 d; the content of the control reached 5.8% at 10 d and then decreased. Ketones and olefins were proportionally the lowest wax components in MT and the control.

### 3.3. Fruit Quality during Storage as Affected by Melatonin Treatment

**Figure 5:**
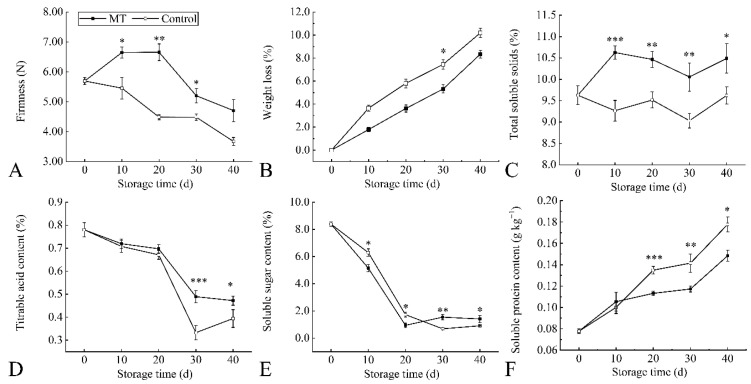
Melatonin treatment affects fruit quality during storage. Changes in firmness (**A**), weight loss (**B**), total soluble solids (**C**), titratable acid content (**D**), soluble sugar content (**E**), and soluble protein content (**F**). Values represent mean ± standard deviation based on three independent biological replicates. * represents *p* < 0.05 for significance between treatments, ** represents *p* < 0.01, *** represents *p* < 0.001.

Peel crispness is a characteristic feature of ‘Kongxin’ plum fruit, and fruit firmness and weight loss are the main factors limiting postharvest quality duration. Compared with the firmness of the control, which decreased continuously, the firmness of MT increased from 0–20 d and decreased to 4.71 N at 40 d (Figure 5A). The weight loss of plum increased during storage. MT was 1.9% lower than the control at 40 d (Figure 5B). Some substances in the fruit were consumed by metabolism during senescence. The TSS and TA contents decreased during storage, but both TSS and TA remained higher in MT (Figure 5C,D). The soluble sugar content of fruit in MT decreased sharply through 20 d and increased in the later stages (Figure 5E). The soluble protein content of plum fruit increased during storage, and the content of MT was 0.15 g·kg^−1^ at 40 d, which was lower than that of the control (Figure 5F).

**Figure 6:**
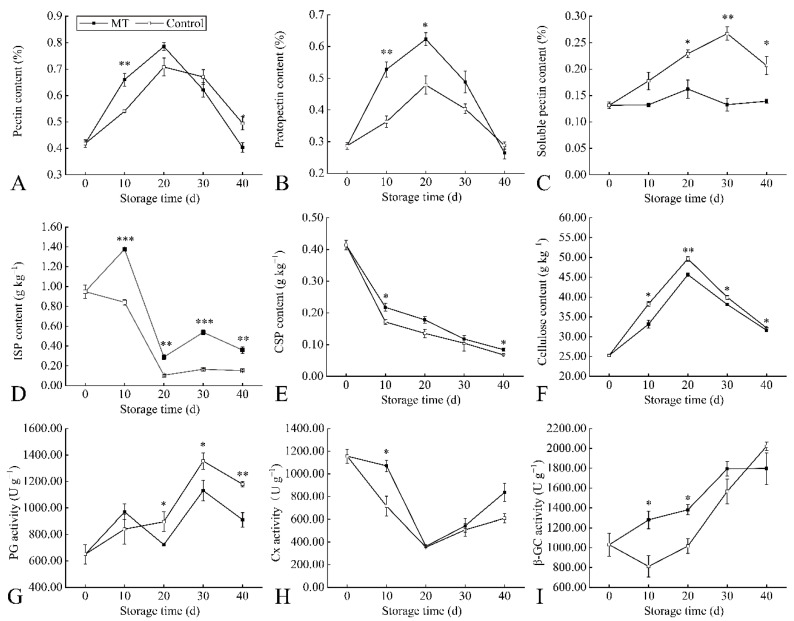
Melatonin treatment affects physiological indexes of fruit during storage. Changes in pectin content (**A**), protopectin content (**B**), soluble pectin content (**C**), ISP content (**D**), CSP content (**E**), cellulose content (**F**), PG activity (**G**), Cx activity (**H**), and β-GC activity (**I**). Values represent mean ± standard deviation based on three independent biological replicates. * represents *p* < 0.05 for significance between treatments, ** represents *p* < 0.01, *** represents *p* < 0.001.

Large quantities of the pectic substance are deposited in the primary wall and mesogel layer of fruit cells and play a vital role in bonding individual cells. PP is formed by the combination of pectin substances and cellulose, which reached the highest level at 20 d, and the pectin content of MT was higher than that of control within 0–20 d (Figure 6A). The content of protopectin in plum fruit during storage was higher than that of soluble pectin. The PP content reached the highest level at 20 d, and the PP content in MT was higher than that in the control. The SP content of MT was lower than that of the control, and the SP content of MT reached the highest at 20 d, while the control was delayed to 30 d (Figure 6B,C). Pectic polymers are crosslinked in different ways to generate various forms of pectins, including ISP and CSP. The content of ISP in plum fruit during storage was higher than that of CSP, and the content of ISP in MT was higher than that of the control. Although CSP content decreased continuously during storage, the levels present in MT were 24.5% higher than that of the control at 40 d (Figure 6D,E). The cellulose content in plum fruit reached the highest level at 20 days and then decreased continuously, while the cellulose content of the control was consistently higher than that of MT (Figure 6F). PG activity in plum fruit reached the highest at 30 d, and PG activity of MT was 16.5% lower than that of the control (Figure 6G). At the same time, the Cx activity of plum fruit was the lowest at 20 d, and the CL activity of MT was 37.4% higher than that of the control at 40 d (Figure 6H). The β-GC activity of the control decreased to the lowest level at 10 d and continued to increase thereafter. Although the β-GC activity of MT increased gradually, the β-GC activity of MT was 11.1% lower than that of the control at 40 d (Figure 6I).

### 3.4. Correlation Analysis of Cuticular Wax Composition and Storage Quality

**Figure 7:**
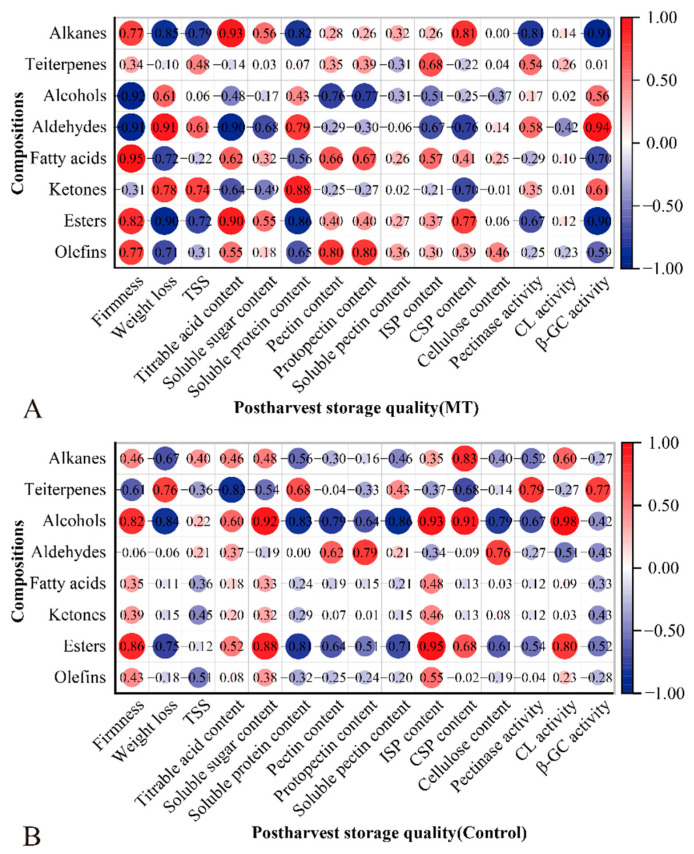
Melatonin treatment affects the storage quality correlation with wax components and alkanes composition. Correlation between wax components and storage quality in MT (**A**) and the control (**B**). Red and blue indicate the positive correlation coefficient and negative correlation coefficient between variables, respectively. |r| > 0.9 represents that correlation was significant.

The correlation between the components of plum cuticular wax of MT and the control and storage quality was analyzed, respectively (Figure 7A,B). Firmness was positively correlated with alcohols (r = 0.82) and esters (r = 0.86) in the control and with fatty acids (r = 0.95) and esters (r = 0.82) in MT. In MT, firmness was negatively correlated with alcohols (r = −0.92) and aldehydes (r = −0.91). In addition, weight loss was correlated with alcohols (r = −0.84) in the control and with alkanes (r = −0.85), aldehydes (r = 0.91), and esters (r = −0.90) in MT. No significant correlations were observed between TSS, pectin, protopectin, cellulose, and PG and the wax components. TA only had a significant correlation with triterpenes (r = −0.83) in the control, and TA was positively correlated with alkanes (r = 0.93) in MT (*p* < 0.01). In the control, soluble sugar significantly correlated with alcohols (r = 0.92) and esters (r = 0.88), and no significant related component was found in MT. Moreover, it was found that soluble protein was correlated with alcohols (r = −0.83) in the control and with alkanes (r = −0.82), ketones (r = 0.88), and esters (r = −0.86) in MT. The results of the correlation analysis showed that soluble pectin, ISP, CSP, and Cx were positively correlated with the alcohols of the control. In addition, only ISP was correlated with ketones in MT, and the other three storage indices had no significant correlation components. However, no component with a significant correlation with β-GC was noted in the control. By contrast, β-GC was negatively correlated with alkanes (r = −0.91) and esters (r = −0.90) and positively correlated with aldehydes (r = 0.94) in MT. Our analysis of the correlation between specific components and storage quality found that the chain length of components affects the correlation with storage quality, especially the alkane composition (Tables S1 and S2). Therefore, the correlation between the alkane composition of MT and the control and storage quality was analyzed, respectively (Figure S1). Melatonin increased the correlation between long-chain alkanes and some storage-quality parameters. The correlation of straight-chain alkanes and their isomers, such as pentadecane with 2, 6, 11-trimethyl-dodecane (C_15_H_32_) and heneicosane with 2, 6, 10, 15-tetramethyl-heptadecane (C_21_H_44_), with storage quality was analyzed. It was found that the difference in the correlation value of the control was higher than that of MT.

## 4. Discussion

This study examined peel microstructure, chemical composition, storage quality, and the relationship between wax components and storage-quality parameters.

Wax bloom was found on plum fruit, and the change of wax bloom was inhibited at low temperatures in the early storage period. This observation was consistent with the fact that low-temperature storage reduced wax metabolism and maintained the surface gloss of pear fruit [29]. Melatonin delayed fruit shrinkage, but the white wax bloom of the control fruit was more evident. The rod-shaped wax structure of the plum gradually disintegrated during storage and then combined with the inner wax. Moreover, the decomposition of wax will decrease the cuticular wax enthalpy of the apple [42,43]. During the storage of blueberry fruit, it was found that the characteristic tubular waxy structure would gradually form block adhesion [34]. Our study found that the rod-shaped wax of melatonin-treated ‘Kongxin’ plum fruit resulted in a coarser wax structure due to the disintegrating crystal but inhibited the combination of disintegrated wax and inner wax.

In previous studies, fruit firmness was closely related to the cell wall. The controlled disassembly of cell-wall polymers contributes to fruit softening, and this process will be accompanied by the depolymerization of pectin and other cell-wall polysaccharides [44,45]. Pectin was considered to be the main component of the primary cell wall. It was reported that fruit softening was accompanied by the degradation of protopectin to soluble pectin [28]. In this study, MT could inhibit the degradation of cell-wall collagen, protect pectin from the influence of hydrolase activity, maintain the normal distribution of pectin, and reduce the soluble pectin content during storage of ‘Kongxin’ plum fruit. The contents of ISP and CSP were negatively correlated with fruit firmness under low-temperature storage, while melatonin promoted the ability of ISP and CSP contents to be positively correlated with fruit firmness. These results suggest that inhibiting the transformation of protopectin to soluble pectin may lead to the delay of melatonin-induced fruit softening, which was consistent with the previous study on the inhibition by valeric acid treatment on the softening of harvested ‘Waizuili’ plum [46].

As the primary component of the plant cell wall, cellulose, together with pectin, maintains the mechanical strength of plant tissue [47]. In this study, cellulose content was negatively correlated with fruit firmness under low-temperature storage, but melatonin promoted a positive correlation between cellulose content and fruit firmness. Although low-temperature storage increased the cellulose content of fruit in the early stage of storage, melatonin promoted a loss in cellulose content. This observation was inconsistent with previous reports that the cellulose content was positively correlated with fruit firmness, that the cellulose content of fruit decreases continuously under room temperature storage, and taht 1-MCP increases the cellulose content of plum fruit [5]. Although the changes in cellulose content and cellulase activity were inconsistent with those reported for room-temperature storage [5], in the present study, the cellulose content decreased with the decrease in fruit firmness in the later stages of storage, indicating that there was a positive correlation between fruit firmness and cellulose content.

PG, Cx, and β-GC play essential roles in the decomposition of cell-wall polysaccharides and participate in the cell-wall remodeling associated with fruit ripening. Melatonin inhibited the decomposition of pectin by reducing the activity of PG, which was consistent with the inhibition of pectin decomposition of the ‘Waizuili’ plum by valeric acid at room temperature [46]. Furthermore, melatonin increased Cx and β-GC activity, and fruit firmness was correlated with β-GC under low-temperature storage. Low-temperature storage is an effective measure to delay fruit softening, but it usually accelerates fruit softening after low-temperature storage. The storage-quality parameters related to fruit firmness under low-temperature storage were soluble sugar and protein. In response to melatonin, however, no significant relationship was found between fruit firmness and storage-quality parameters. This observation indicates that melatonin can effectively maintain fruit firmness and reduce the correlation between fruit firmness and other storage-quality parameters (Tables S1 and S2).

Cuticular wax is the first barrier to protect the fruit from biotic and abiotic stresses and helps to maintain fruit firmness and delay fruit softening [48,49]. Waxes affect the chemical and mechanical properties of fruit cell walls and further affect the changes in firmness [50]. Crispness is a significant feature of ‘Kongxin’ plum fruit, and fruit firmness and weight loss are the main factors limiting the shelf life. In this study, there was a significant negative correlation between fruit firmness and weight loss under low-temperature storage (*p* < 0.01), while fruit softening and weight loss was inhibited with melatonin, and the correlation between firmness and weight loss was reduced. Nineteen wax components were correlated with firmness in MT, while only 13 were correlated with firmness in the control. Esters were the most numerous compositions correlated with firmness in the control. Fatty acids were the most abundant cuticular component, which correlated significantly with firmness in MT. In the correlation analysis with various components, firmness was positively correlated with esters in the control and correlated with fatty acids in MT. Alkanes in plum peel wax were positively correlated with firmness, which was inconsistent with the negative correlation between alkanes and firmness during pear storage. However, MT promoted a negative correlation between short-chain alkanes and firmness. In addition, there was a positive correlation between alkanes and esters in plum-peel wax, which was consistent with pear storage [29]. The total component only explains the correlation with storage indices to a certain extent. The correlation between specific compositions and storage indices can better explain the correlation between components and storage indices.

Most naturally occurring alkanes do not exceed 50 carbons. With increasing chain length, alkanes are inherently more hydrophobic and are increasingly unstable and prone to degradation. In our study, the analyzed alkane carbon chain length was between 12 and 36. Melatonin-promoted long-chain alkanes were positively correlated with firmness and water retention and made the correlation between the length of the alkane chain and storage-quality parameters more obvious, but reduced the difference between alkane isomers and storage-quality parameters. According to the group addition method proposed by Benson et al. [36], branched alkanes are more stable than straight alkanes. They have higher hydrophobicity, whereas the synthesis of branched alkanes required more energy, resulting in straight alkane content in peel wax being much higher than branched alkane content. Furlan et al. [51] observed that air pollutants promote an increase in the content of short-chain alkanes (C19-C23) and a decrease in long-chain alkanes (C24-C31), indicating that pollutants promote the synthesis of more short-chain hydrocarbons. In addition, it has been reported that cuticle components’ weighted average chain length may be negatively correlated with water permeability [52]. In a study of the effect of a high concentration of thrips-susceptible pepper accessions (*Capsicum* spp.) on leaf wax alkanes, it was found that very long-chain wax alkanes played an essential role in the thrips susceptibility of pepper [53].

## 5. Conclusions

The characteristic rod-shaped waxy structure of plum disintegrated during storage. Melatonin promoted the disintegration and thickening of rod-shaped waxy crystals of ‘Kongxin’ plum fruit but inhibited the combination of disintegrated wax and inner wax. Melatonin inhibited the degradation of protopectin to soluble pectin by decreasing PG activity, and the content of soluble pectin in ‘Kongxin’ plum fruit treated with melatonin was kept at a low level. In addition, melatonin increased the activities of Cx and β-GC and delayed the softening of ‘Kongxin’ plum fruit so that the fruit could maintain a certain degree of firmness during storage, thus prolonging the storage time.

## Data Availability

Data are presented within the article.

## Supplementary Materials

The following supporting information can be downloaded at: https://www.mdpi.com/article/10.3390/foods11243972/s1, Table S1 Correlation between storage quality parameters and wax components in MT. Table S2 Correlation between storage quality parameters and wax components in the control. Figure S1. Effect of melatonin treatment on the storage quality correlation with wax components and alkanes composition.
