# Supplementary material for: Melatonin Treatment Affects Wax Composition and Maintains Storage Quality in ‘Kongxin’ Plum (Prunus salicina L. cv) during Postharvest
Source: Foods. 2022 Dec 8;11(24):3972. doi: 10.3390/foods11243972 (PMC9778571; doi:10.3390/foods11243972)
Supplement: Supplementary file 1 [file foods-11-03972-s001.zip › Figure S1.pdf]

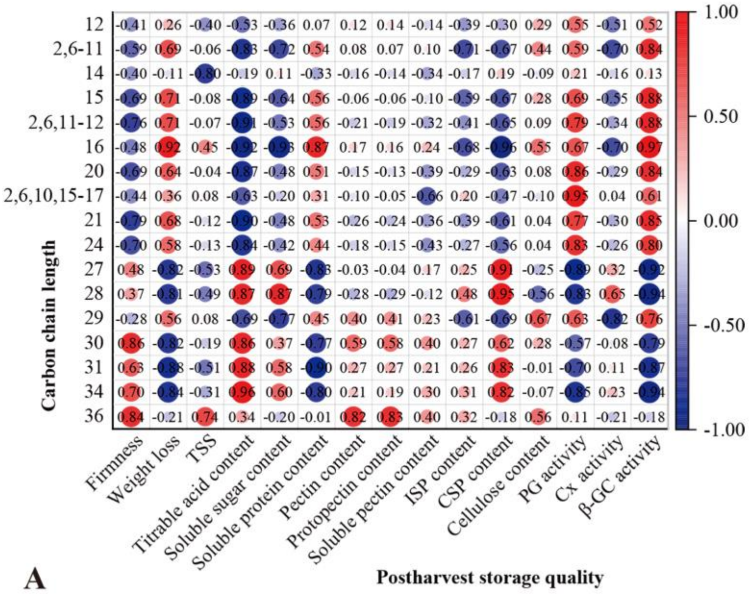

A

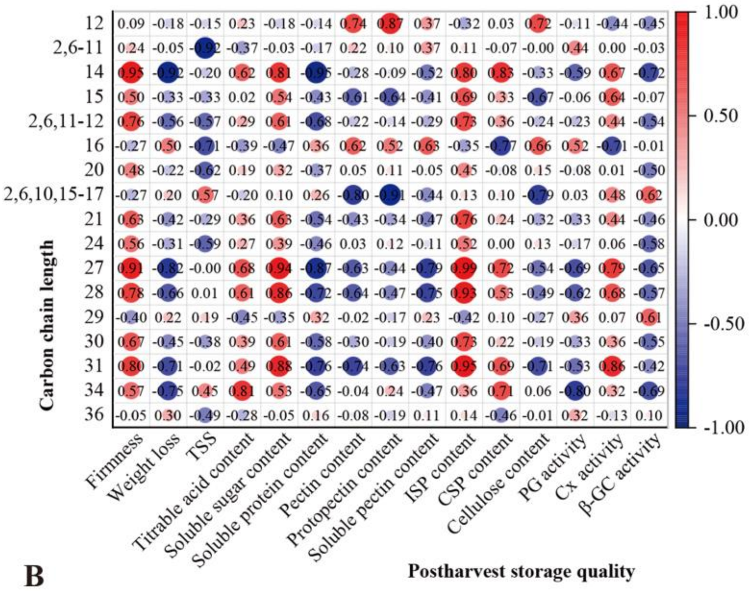

B

Figure S1. Effect of melatonin treatment on the storage quality correlation with wax components and alkanes composition. Correlation between alkanes composition and storage quality in MT **(A)** and control **(B)**. Red and blue indicate the positive correlation coefficient and negative correlation coefficient between variables respectively.
